# Supplementary material for: Antimicrobial resistance dissemination via horizontal gene transfer is constrained in stratified waters
Source: Commun Biol. 2026 Mar 12;9:435. doi: 10.1038/s42003-026-09857-8 (PMC13022341; doi:10.1038/s42003-026-09857-8)
Supplement: Supplementary file 2 — Supplementary Information [file 42003_2026_9857_MOESM2_ESM.pdf]

Supplementary Material for:
Antimicrobial resistance dissemination via horizontal gene transfer is constrained in stratified waters

Máté Vass[1,2,3*], Anna Abramova[1,2], Johan Bengtsson-Palme[1,2,4]

[1]Division of Systems and Synthetic Biology, Department of Life Sciences, Science for Life Laboratory, Chalmers University of Technology, SE-412 96, Gothenburg, Sweden
[2]Centre for Antibiotic Resistance Research (CARe) in Gothenburg, Sweden
[3]Division of Microbial Ecology, Department of Aquatic Sciences and Assessment, Swedish University of Agricultural Sciences, SE-756 51, Uppsala, Sweden
[4]Department of Infectious Diseases, Institute of Biomedicine, The Sahlgrenska Academy, University of Gothenburg, SE-413 46, Gothenburg, Sweden

*corresponding author: mate.vass@slu.se

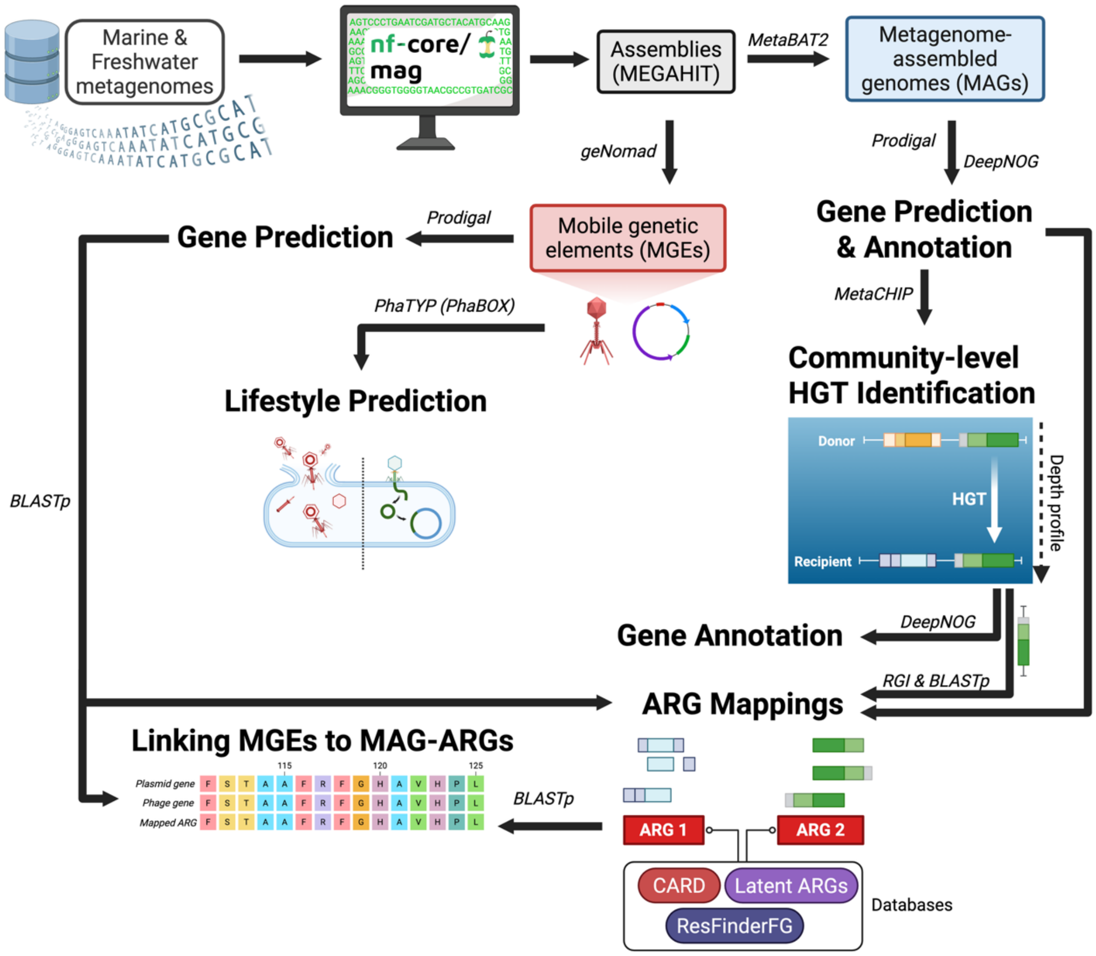

Supplementary Figure 1. Overview of the data processing and analysis pipeline.

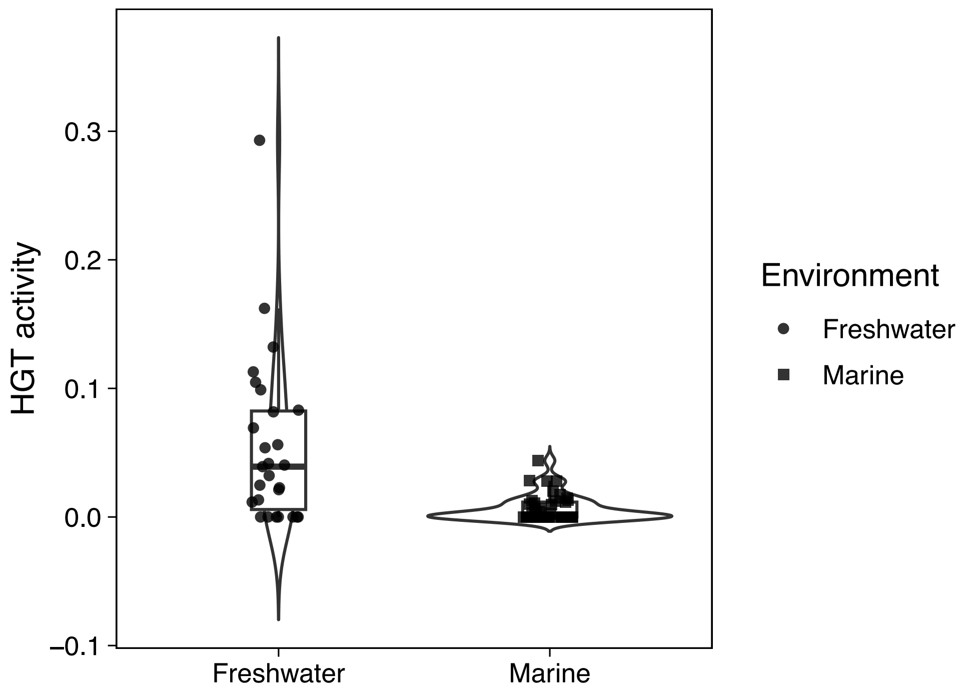

Supplementary Figure 2. Distribution of HGT activity in freshwater and marine environments. Boxplots show the interquartile range (IQR), median and whiskers (1.5× IQR).

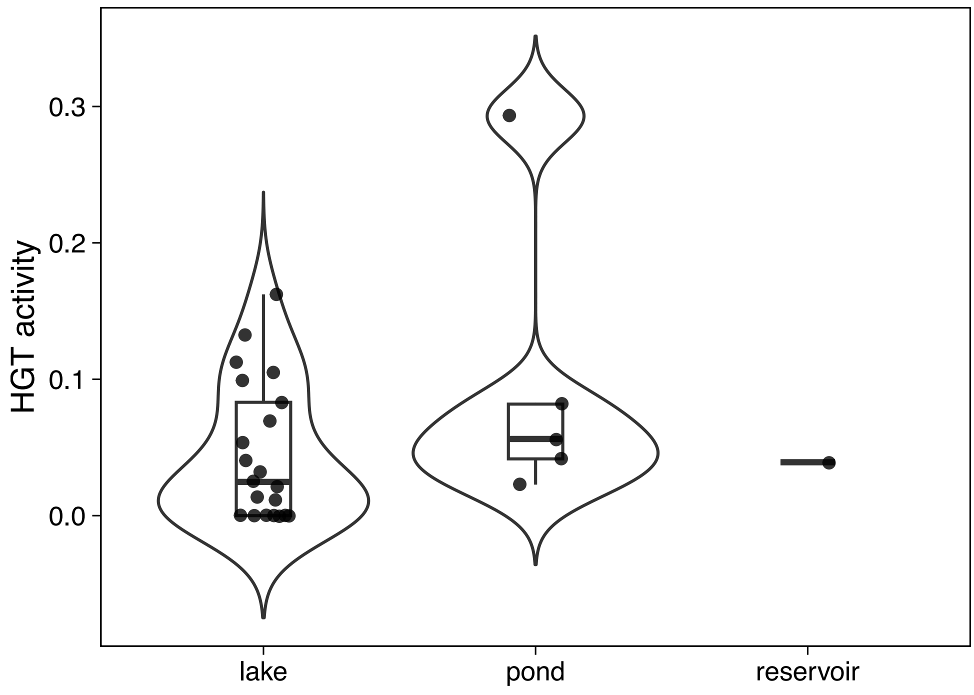

Supplementary Figure 3. Distribution of HGT activity within freshwater environments. Boxplots show the interquartile range (IQR), median and whiskers (1.5× IQR).

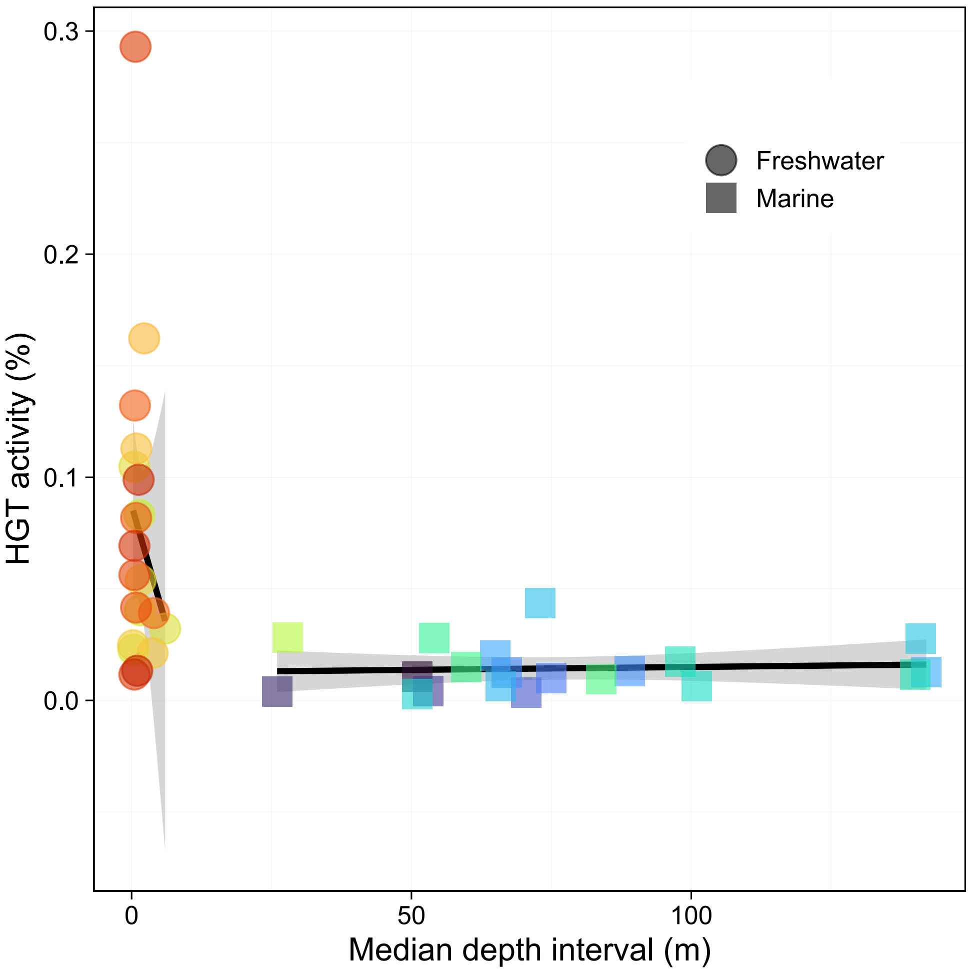

Supplementary Figure 4. Median depth interval (m) in each sampling location in the presence of horizontal gene transfer (HGT). HGT activity refers to the proportion of HGT-mediated unique genes, expressed as a percentage of the total number of genes within a sampling location. Black lines refer to regression lines per environment with standard error.

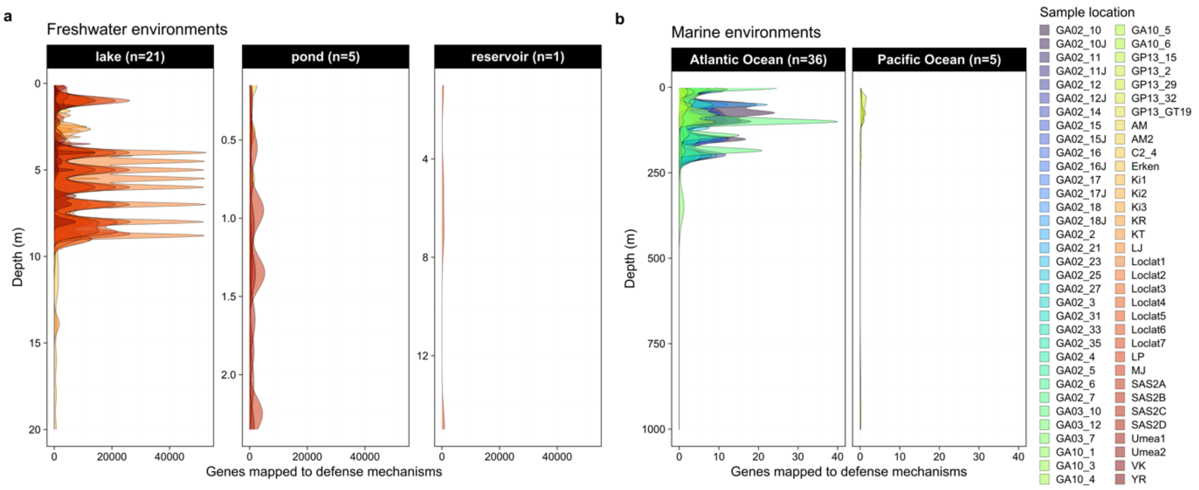

Supplementary Figure 5. Vertical distribution of predicted functional genes based on open reading frames (ORFs) associated with defence mechanisms (COG category 'V'), as proxy for the presence of stressors.

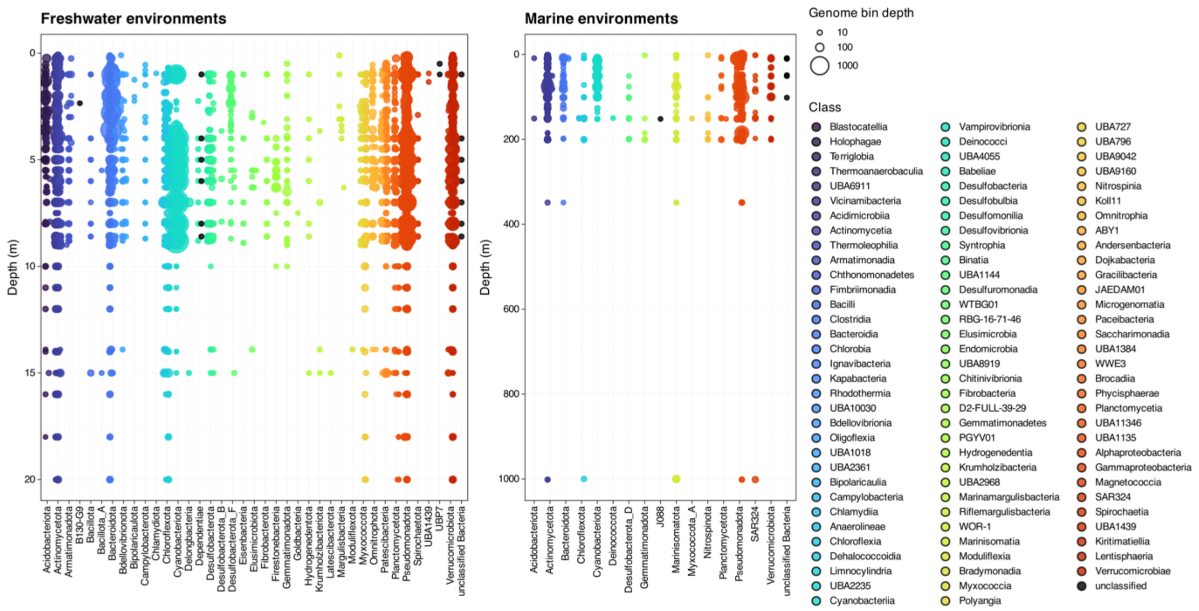

Supplementary Figure 6. Vertical distribution of microbial taxa in the analysed freshwater (n = 27) and marine (n = 41) environmental samples, based on metagenome-assembled genomes.

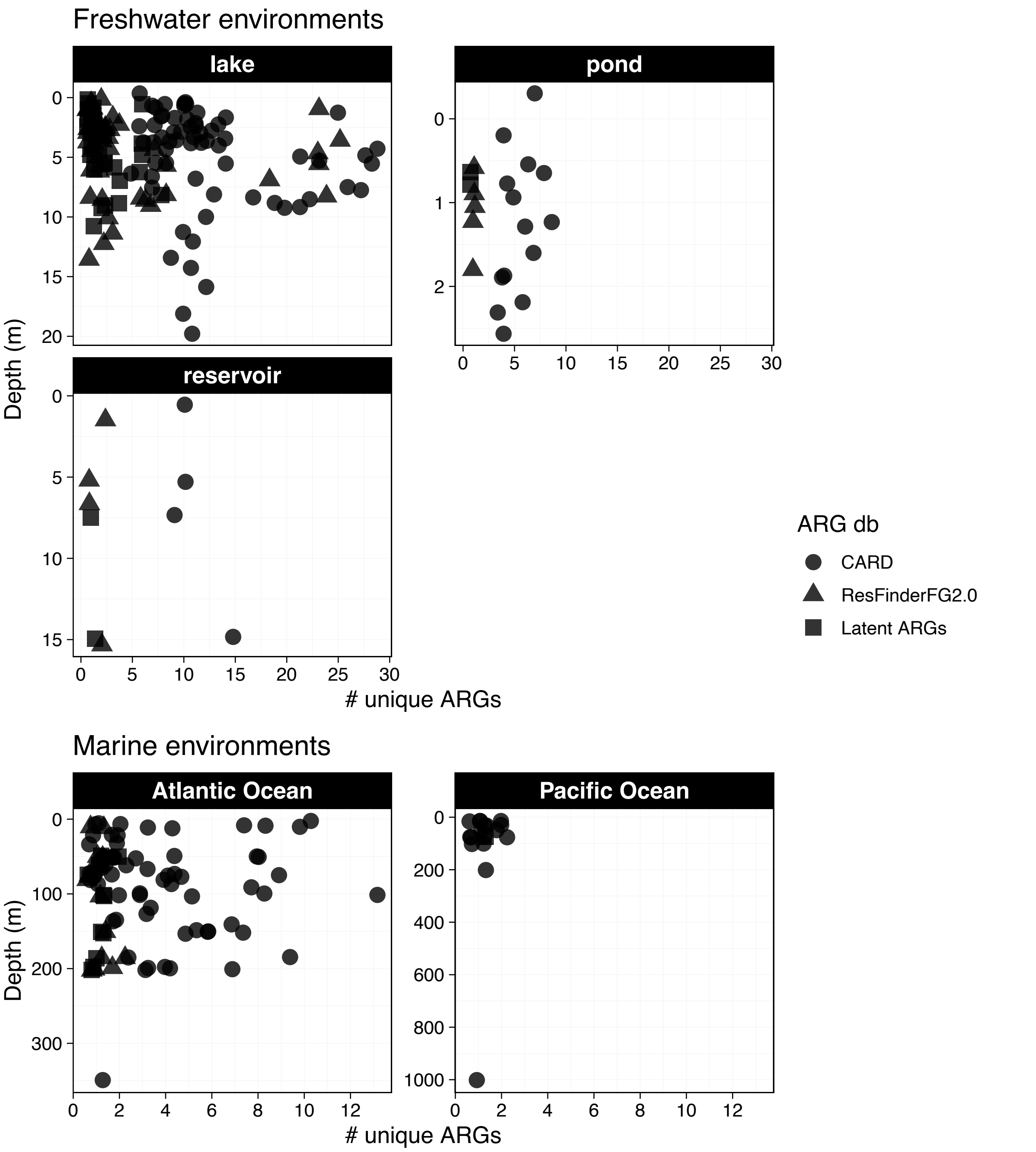

Supplementary Figure 7. Spatial distribution of distinct antibiotic resistance genes (ARGs) across water depths in various freshwater, such as lake (n=21), pond (n=5) and reservoir (n=1), as well as in marine (Atlantic Ocean: n=36, Pacific Ocean: n=5) sites. Symbols denote the three reference databases used in this study to identify ARGs: for the identification of well-known (established) ARGs, we utilised the Comprehensive Antibiotic Resistance Database (CARD)(Alcock et al., 2023) and the ResFinderFG2.0(Gschwind et al., 2023) database, while for the detection of computationally predicted (latent) ARGs, a database compiled by (Inda-Diaz et al., 2023) was referenced.

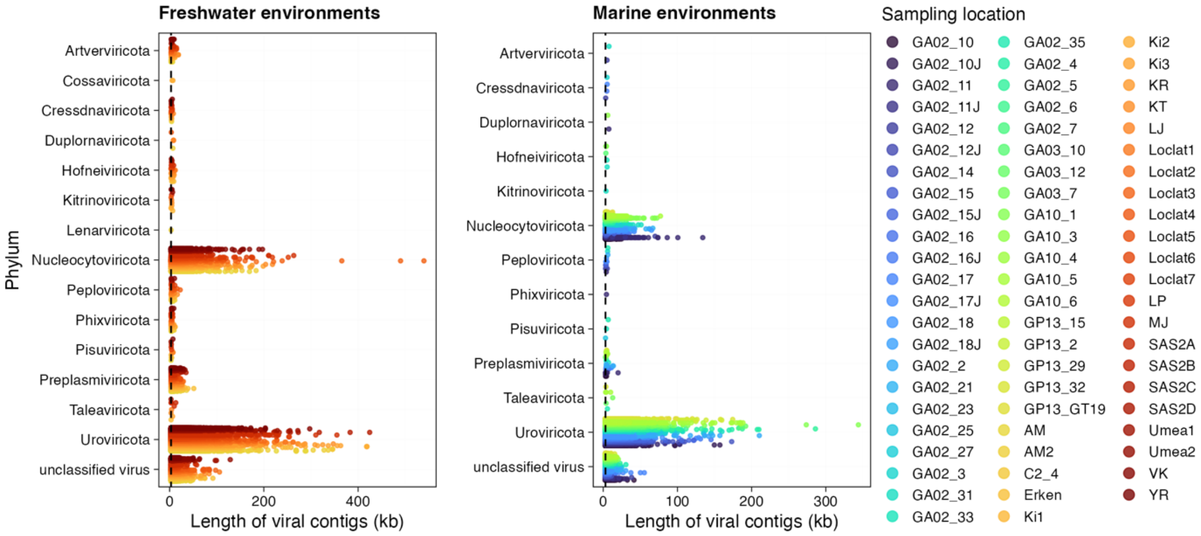

Supplementary Figure 8. Recovered viral contigs by geNomad in freshwater (n = 27) and marine (n = 41) environmental samples. Dashed line indicate our cutoff level of 3,000 bp in contig length. For details on Sample location, see, Supplementary Table 8, 9.

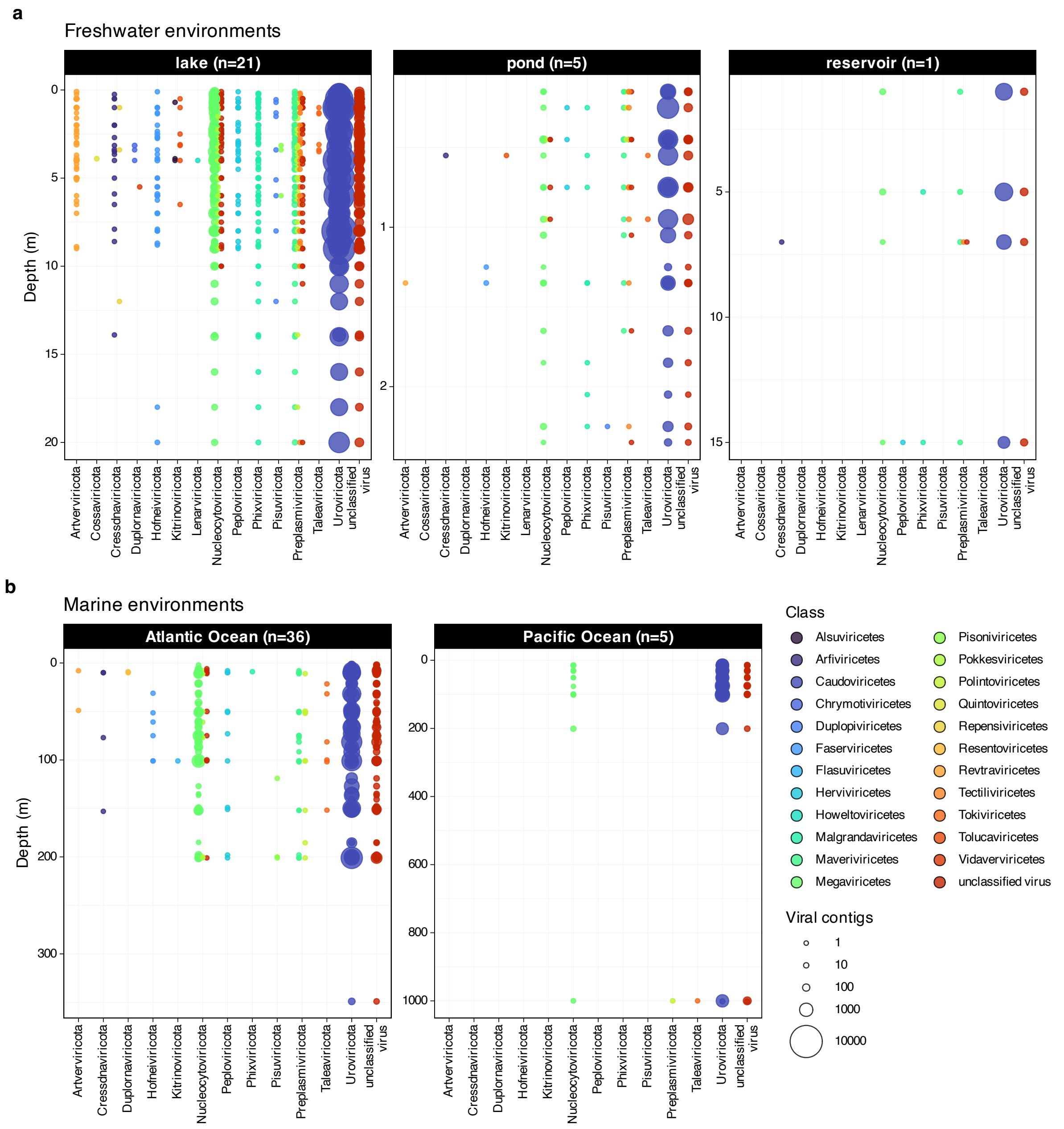

Supplementary Figure 9. Vertical distribution of recovered viral contigs in freshwater (n = 27) and marine (n = 41) samples. Viral contigs were taxonomically annotated by geNomad.

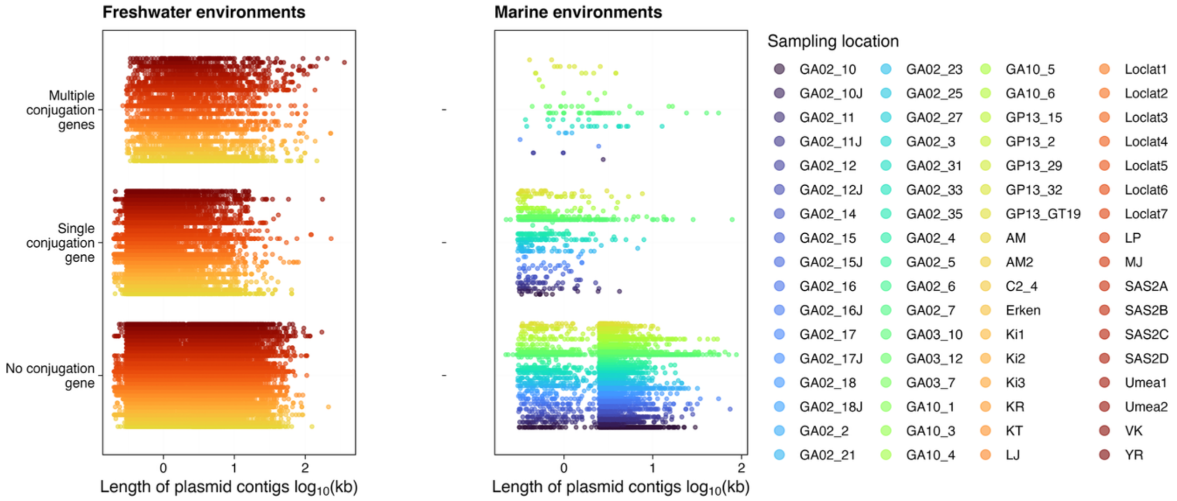

Supplementary Figure 10. Recovered plasmid contigs by geNomad in freshwater (n = 27) and marine (n = 41) environmental samples. For details on Sample location, see, Supplementary Table 8, 9.

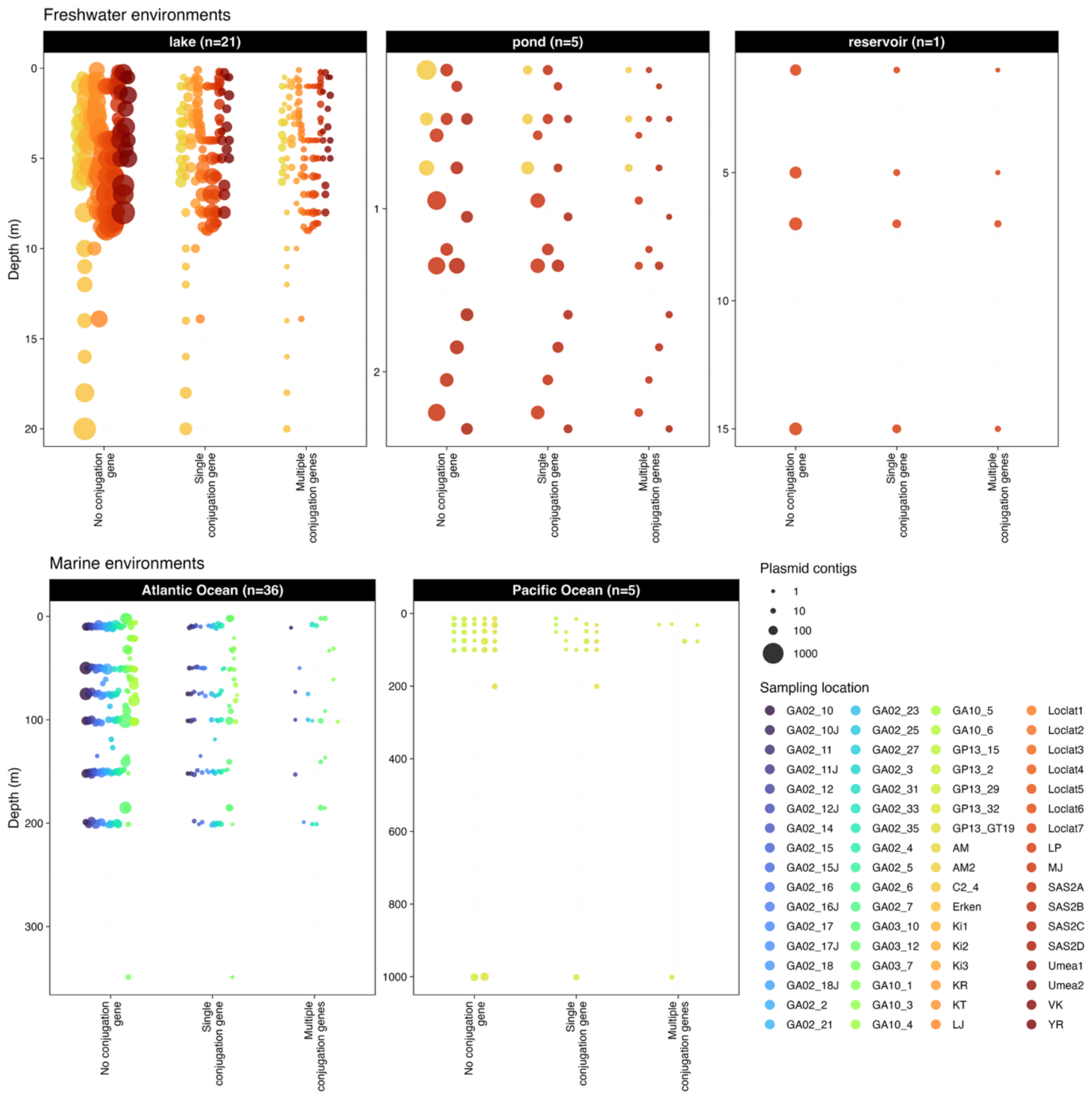

Supplementary Figure 11. Vertical distribution of plasmids in freshwater (n = 27) and marine (n = 41) sample locations. Plasmid contigs were identified by geNomad. For details on Sample location, see, Supplementary Table 8, 9.

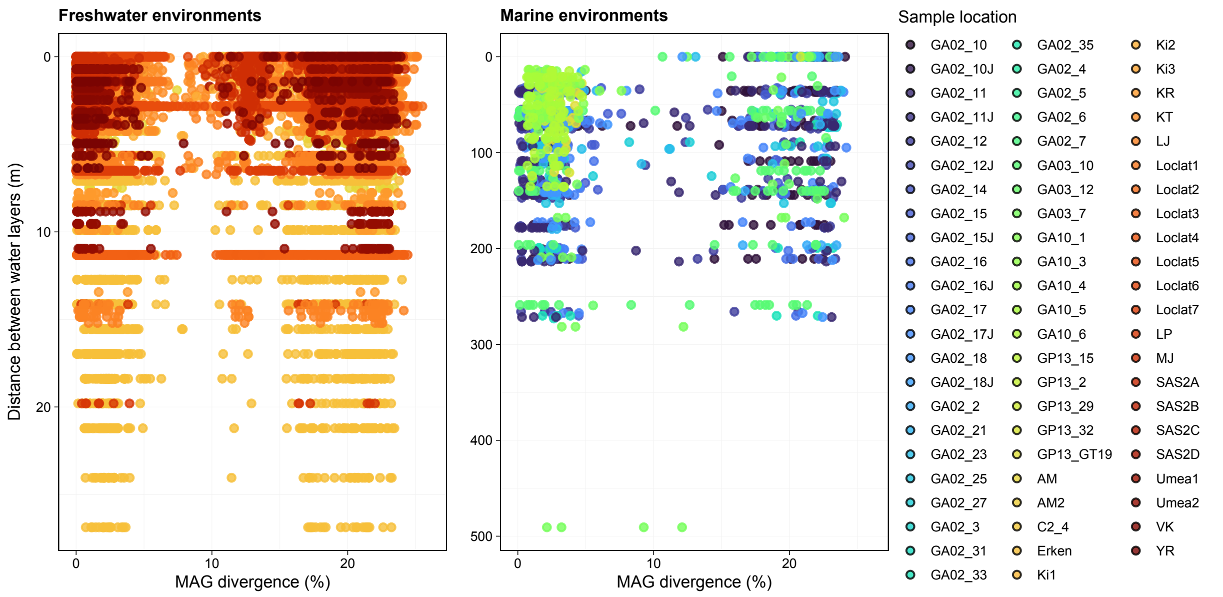

Supplementary Figure 12. Sequence similarity of metagenome-assembled genomes (MAGs), based on calculated average nucleotide similarity by FastANI. For details on Sample location, see, Supplementary Table 8, 9.

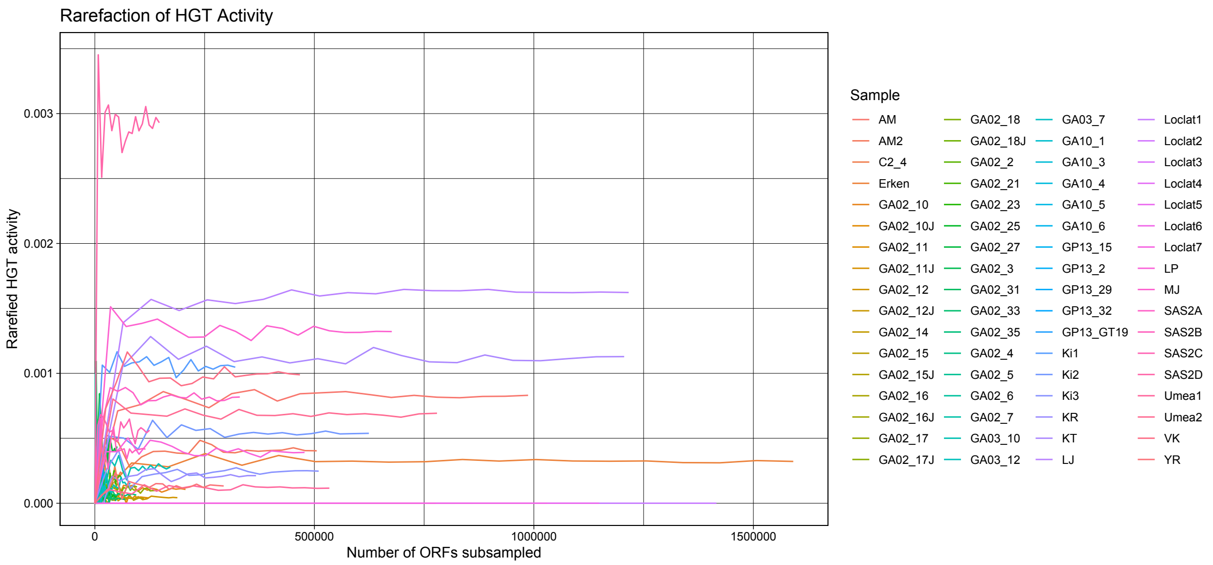

Supplementary Figure 13. Rarefaction curves of ORFs demonstrating the HGT activity rapidly stabilized with increasing sampling effort.

References

Alcock, B. P., Huynh, W., Chalil, R., Smith, K. W., Raphenya, A. R., Wlodarski, M. A., Edalatmand, A., Petkau, A., Syed, S. A., Tsang, K. K., Baker, S. J. C., Dave, M., McCarthy, M. C., Mukiri, K. M., Nasir, J. A., Golbon, B., Imtiaz, H., Jiang, X., Kaur, K.,…McArthur, A. G. (2023). CARD 2023: expanded curation, support for machine learning, and resistome prediction at the Comprehensive Antibiotic Resistance Database. *Nucleic Acids Res*, *51*(D1), D690–D699. https://doi.org/10.1093/nar/gkac920

Gschwind, R., Ugarcina Perovic, S., Weiss, M., Petitjean, M., Lao, J., Coelho, L. P., & Ruppe, E. (2023). ResFinderFG v2.0: a database of antibiotic resistance genes obtained by functional metagenomics. *Nucleic Acids Res*, *51*(W1), W493–W500. https://doi.org/10.1093/nar/gkad384

Inda-Diaz, J. S., Lund, D., Parras-Molto, M., Johnning, A., Bengtsson-Palme, J., & Kristiansson, E. (2023). Latent antibiotic resistance genes are abundant, diverse, and mobile in human, animal, and environmental microbiomes. *Microbiome*, *11*(1), 44. https://doi.org/10.1186/s40168-023-01479-0
