# Supplementary material for: Antimicrobial resistance dissemination via horizontal gene transfer is constrained in stratified waters
Source: Commun Biol. 2026 Mar 12;9:435. doi: 10.1038/s42003-026-09857-8 (PMC13022341; doi:10.1038/s42003-026-09857-8)
Supplement: Supplementary file 3 — Description of Additional Supplementary Files [file 42003_2026_9857_MOESM3_ESM.pdf]

Description of Additional Supplementary File

File name: Supplementary data 1-5
Description: Source data underlying the figures can be found in supplementary data 1-5.

File name: Supplementary data 6
Description: Horizontal gene transfers across sampled water layers

File name: Supplementary data 7
Description: Taxonomy of marine MAGs with >40% completeness

File name: Supplementary data 8
Description: Taxonomy of freshwater MAGs with >40% completeness

File name: Supplementary data 9
Description: Distribution of HGT-mediated genes across COG categories

File name: Supplementary data 10
Description: Identified MAG-ARGs at various depths by three ARG databases (see 'Method' column). 'Bin_depth' column represents MAG abundances from contig sequencing depths.

File name: Supplementary data 11
Description: Number of identified antibiotic resistance genes (ARGs), ARGs mediated by horizontal gene transfers (HGTs) across water depths, and the total number of estimated HGTs.

File name: Supplementary data 12
Description: Genes identified as ARGs by more than a single database (see 'Method' column). 'Bin_depth' column represents MAG abundances from contig sequencing depths.

File name: Supplementary data 13
Description: Genes on viral MGE contigs (query) mapped to MAG-ARGs (target)

File name: Supplementary data 14
Description: Sequence similarity (ANI) of freshwater MAGs

File name: Supplementary data 15
Description: Sequence similarity (ANI) of marine MAGs

File name: Supplementary data 16
Description: Sample list from freshwater environments. For further details, please see: https://doi.org/10.1038/s41597-021-00910-1

File name: Supplementary data 17
Description: Sample list from marine environments. For further details, please see: https://doi.org/10.1038/sdata.2018.176

File name: Supplementary data 18
Description: Mixed-layer depth calculated for marine sampling sites.
